# Osimertinib Resistance With a Novel *EGFR* L858R/A859S/Y891D Triple Mutation in a Patient With Non-Small Cell Lung Cancer: A Case Report

**DOI:** 10.3389/fonc.2020.542277

**Published:** 2020-11-26

**Authors:** Yanli Yang, Xing Zhang, Ruixiao Wang, Jiayue Qin, Juan Wang, Zhimin Li, Xia Song

**Affiliations:** ^1^ Department of Respiration Medicine, Shanxi Cancer Hospital, Taiyuan, China; ^2^ Department of Medical Affairs, Annoroad Gene Technology Co., Ltd, Beijing, China; ^3^ Department of Research and Development, Annoroad Institute of Life Sciences, Jinhua, China

**Keywords:** case report, osimertinib, epidermal growth factor receptor, circulating tumor DNA, non-small cell lung cancer

## Abstract

Targeted drug therapy based on the types of epidermal growth factor receptor (*EGFR*) gene mutations has been widely used in the diagnosis and treatment of patients with non-small cell lung cancer (NSCLC). With the development of next-generation sequencing (NGS) technology, more and more *EGFR*-tyrosine kinase inhibitor (TKI) resistance mutation sites have been revealed. Here, we report a novel *EGFR* L858R/A859S/Y891D triple mutation in plasma-derived circulating tumor DNA (ctDNA) was identified in a 53-year-old male patient with NSCLC resistant to osimertinib treatment, using an ultra-deep (20,000×) 160-gene panel through the NGS platform. Our case confirms that dynamic monitoring of liquid biopsy based on ctDNA is conducive to the selection of targeted therapy and the realization of the patient’s full course management.

## Introduction

Accurate identification of oncogenic driver mutations has revolutionized the clinical management of non-small cell lung cancer (NSCLC). Targeted drug therapy based appropriate epidermal growth factor receptor (*EGFR*) gene mutations has been widely used in the treatment of NSCLC patients (1, 2). Based on the kinase domain of *EGFR*, several *EGFR*-tyrosine kinase inhibitor (TKI) drugs have been developed and applied effectively, including the first-generation inhibitors erlotinib, gefitinib and icotinib, the second-generation inhibitors afatinib and dacomitinib, and the third-generation inhibitor osimertinib (3–8). Although targeted therapy has improved the prognosis of NSCLC patients, inevitable drug resistance remains widespread (9).

With the rapid development of next-generation sequencing (NGS) technology, more and more novel *EGFR* mutation sites have been revealed gradually, indicating the sensitivity and resistance to drugs (10, 11). The biological simulation of protein structure suggested that the first-generation TKI resistance of a patient with NSCLC harboring *EGFR* L858R mutation treated with erlotinib was related to the secondary *EGFR* Y891D mutation (12).

Here, we report a novel *EGFR* L858R/A859S/Y891D triple mutation in plasma-derived circulating tumor DNA (ctDNA) was identified in a patient with NSCLC resistant to osimertinib treatment, using an ultra-deep (20,000×) 160-gene panel through the NGS platform. We present the following case in accordance with the CARE Guideline (13).

## Case Presentation

**Figure 1:**
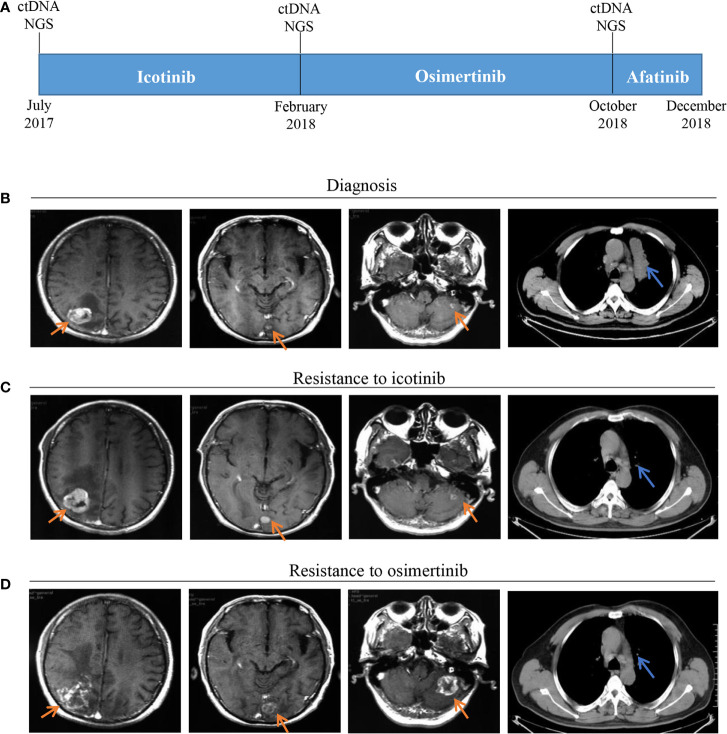
Timeline and effect of *EGFR*-TKI treatments. **(A)** Treatment timeline; **(B)** Baseline images of CT and MRI at diagnosis; **(C)** Brain metastases progressed and original lung mass reduced after seven months of icotinib treatment; **(D)** Brain metastases progressed and thoracic lesions controlled after eight months of osimertinib treatment; EGFR, epithelial growth factor receptor; TKI, tyrosine kinase inhibitor; CT, computed tomography; MRI, magnetic resonance imaging.

**Figure 2:**
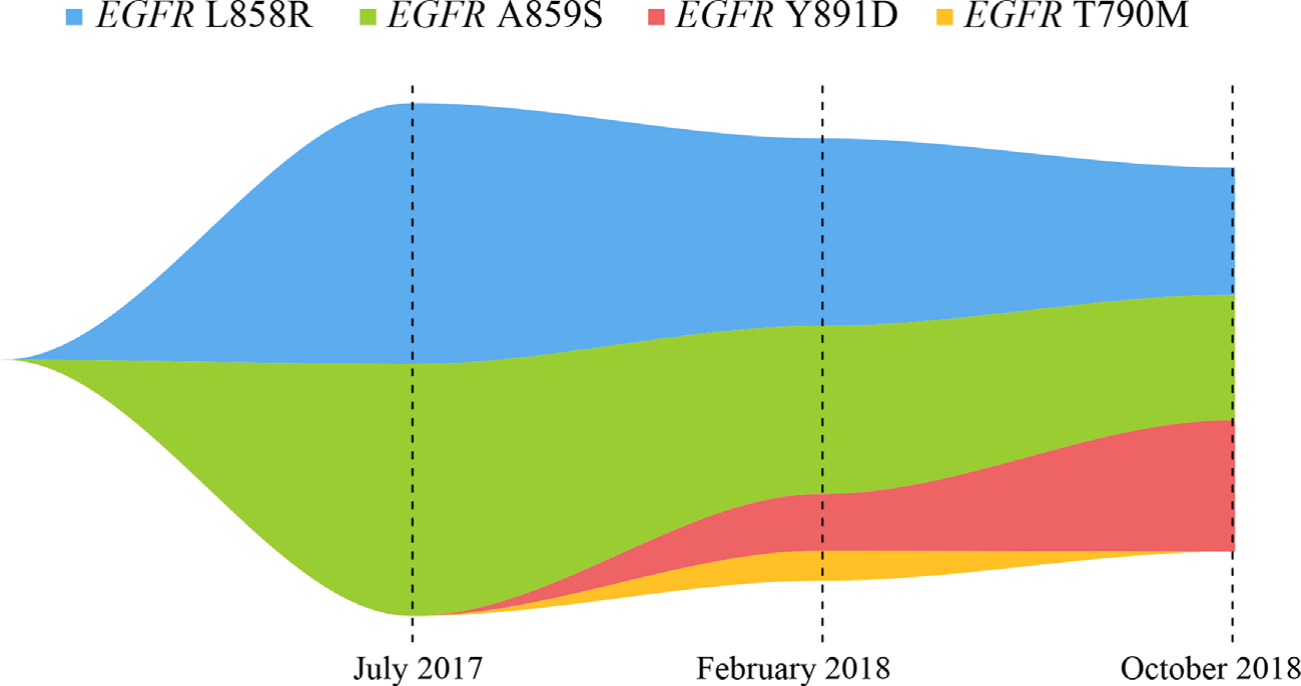
Map of clonal evolution. The different colors represent different mutation sites in *EGFR* gene, as measured by the NGS platform; NGS, next-generation sequencing.

A 53-year-old male with a history of smoking for approximately 30 years was presented to hospital in July 2017. The patient was previously in good health and had no history of other diseases or medication. Computed tomography (CT) scans showed a lung mass of the upper left lobe along with nodules involvement (**Figure 1B**). Brain magnetic resonance imaging (MRI) revealed brain metastases (**Figure 1B**). Broncho-alveolar lavage fluid (BALF) confirmed squamous cell carcinoma. He was diagnosed with stage IVb lung squamous cell carcinoma (T4N2M1c) with a *EGFR* L858R/A859S double mutation, detected using ctDNA through the NGS platform. Variant allele frequencies (VAFs) of the detected *EGFR* L858R and A859S mutations were 8.7 and 8.41%, respectively (**Figures 1A**, **2**). Icotinib (125 mg, three times per day) was then administered from July 2017.

After seven months of icotinib treatment, the patient presented obvious nausea and vomiting, accompanied by lack of consciousness and limited movement of legs. Chest CT scans showed original lung mass was significantly reduced, but the brain MRI showed the brain metastases was enlarged according to the Response Evaluation Criteria in Solid Tumors (RECIST v1.1) (**Figure 1C**). These phenomena can be inferred that the patient had developed resistance to icotinib. Plasma ctDNA detection results showed two secondary *EGFR* T790M and Y891D mutations, and VAFs were 1 and 1.89% respectively, accompanied with decreased VAFs of *EGFR* L858R and A859S mutations to 6.25 and 5.61%, respectively (**Figures 1A**, **2**). Based on ctDNA testing results, the patient received osimertinib (80 mg per day) and experienced significant improvements in nausea, vomiting, consciousness and legs movement within one week. In our subsequent follow-up, the symptoms of the patient were gradually alleviated and the patient was able to take care of himself normally. This good performance status lasted for eight months, suggesting that the symptoms of brain metastasis were well controlled.

However, the disease progressed again after eight months, and the patient presented the neurological symptoms again. Chest CT scans showed thoracic lesions were still well controlled, but brain MRI revealed the brain metastases were larger and the edema was more obvious than before (**Figure 1D**). Compared with the VAFs of *EGFR* mutation sites in the second ctDNA testing, the third gene detection results revealed *EGFR* T790M disappeared, L858R and A859S decreased to 4.25 and 4.18% respectively, while Y891D increased to 4.38% (**Figures 1A**, **2**). The patient received afatinib (40 mg per day) and experienced improvements in nausea, vomiting, consciousness and legs movement within two weeks.

The symptom was well controlled for nearly two months. Eventually, the patient’s condition deteriorated dramatically, resulting in loss of consciousness and paralysis of both legs. His death was certified at home in December 2018, most likely due to the stroke caused by the brain metastases.

## Discussion

Patients with NSCLC harboring *EGFR* mutations are usually treated with *EGFR*-TKIs for targeted therapy, but some patients’ progress was due to acquired drug resistance. *EGFR* mutations are rare in patients with the squamous cell carcinoma, whose outcomes are usually inferior to *EGFR*-positive adenocarcinoma. To date, more and more reports have been published on the mechanisms of third-generation *EGFR*-TKI acquired resistance, such as gatekeeper *EGFR* C797S mutation, human epidermal growth factor receptor 2 (HER2) or hepatocyte growth factor receptor (MET) amplification, and histological transformation (9, 14). Therefore, considering the importance of gene-guided therapy and the difficulty of repeated biopsy or insufficient tissues during the progression, dynamic monitoring of gene mutation variations through liquid biopsy is of great value for the management of NSCLC.

Here, we report a case of whole-course management in a patient with NSCLC carrying *EGFR* gene mutations in ctDNA through the NGS platform, to guide drug selections for *EGFR*-TKI treatments. The NGS detection platform included the detection of gene amplifications, but no gene amplification was found in our study. Previous studies have reported that a patient with *EGFR* L858R/A859S responded well to the first-generation *EGFR*-TKI, which was consistent with our findings (15). Qin et al. inferred that *EGFR* L858R/Y891D was resistant to erlotinib through the energy simulation of protein structure biology and the clinical manifestations of the patient, and found that osimertinib treatment could control the disease status (12). Our present study found that the *EGFR* L858R/A859S/Y891D triple mutation showed drug resistance to osimertinib, so it was concluded that the triple mutation might be the drug resistance mechanism of the patient. In our report, this patient developed the common *EGFR* T790M drug-resistant mutation and the rare *EGFR* Y891D mutation after seven months of treatment with icotinib. Although VAFs of *EGFR* T790M, L858R and A859S were all expected to decrease, VAF of *EGFR* Y891D was significantly increased after osimertinib treatment for eight months (**Figure 2**). The clinical significance of single *EGFR* A859S somatic mutation was not clearly determined, this site was only detected in multiple myeloma, but not reported in lung cancer (16). Combined with these data, we concluded that the secondary *EGFR* Y891D mutation may be the main cause of drug resistance to osimertinib. This phenomenon may be caused by the selection pressure of different *EGFR*-TKI drugs, and the resistance to treatments may be caused by the expansion of the pre-existing subclonal population (17).

It has been reported that patients with rare *EGFR* mutations may be sensitive to targeted treatment with afatinib (18). In this case study, since the *EGFR* A859S and Y891D were considered to be rare *EGFR* mutations, the patient was treated with afatinib for the third-line therapy. Although the symptoms were relieved for nearly two months, the patient died from a stroke caused by brain metastases.

There are some limitations in our study. First of all, the osimertinib resistance mechanism of the novel *EGFR* L858R/A859S/Y891D triple mutation needs to be further verified from the in vitro cell line experiments and protein structural biology energy calculation. Secondly, whether afatinib can be used for the treatment of such patients with this triple mutation remains to be further studied.

## Conclusion

In summary, our report indicates that a novel *EGFR* L858R/A859S/Y891D formed by secondary *EGFR* Y891D may be the potential cause of the drug resistance mechanism of the first- and third-generation *EGFR*-TKIs, which may be a new target for the treatment of NSCLC. In addition, it is confirmed that dynamic monitoring of liquid biopsy based on ctDNA is conducive to the selection of targeted therapy and the realization of the patient’s full course management.

## Ethics Statement

The studies involving human participants were reviewed and approved by Clinical Research Ethics Committee of Shanxi Cancer Hospital. The patients/participants provided their written informed consent to participate in this study. Written informed consent was obtained from the individual(s) for the publication of any potentially identifiable images or data included in this article.

## Author Contributions

All authors contributed to the article and approved the submitted version. YY, XZ, and XS carried out the studies, participated in collecting data, and drafted the manuscript. RW, JQ, JW, and ZL performed the NGS platform and statistical analysis.

## Conflict of Interest

Authors RW, JQ, JW and ZL were employed by the company Annoroad Gene Technology Co., Ltd.

The remaining authors declare that the research was conducted in the absence of any commercial or financial relationships that could be construed as a potential conflict of interest.

## References

[1] Rosell R Moran T Queralt C Porta R Cardenal F Camps C Screening for epidermal growth factor receptor mutations in lung cancer. N Engl J Med (2009) 361(10):958–67. 10.1056/NEJMoa0904554 19692684

[2] Castellanos E Feld E Horn L Driven by Mutations: The Predictive Value of Mutation Subtype in EGFR-Mutated Non-Small Cell Lung Cancer. J Thorac Oncol (2017) 12(4):612–23. 10.1016/j.jtho.2016.12.014 28017789

[3] Zhou C Wu YL Chen G Feng J Liu XQ Wang C. Erlotinib versus chemotherapy as first-line treatment for patients with advanced EGFR mutation-positive non-small-cell lung cancer (OPTIMAL, CTONG-0802): a multicentre, open-label, randomised, phase 3 study. Lancet Oncol (2011) 12(8):735–42. 10.1016/S1470-2045(11)70184-X 21783417

[4] Maemondo M Inoue A Kobayashi K Sugawara S Oizumi S Isobe H Gefitinib or chemotherapy for non-small-cell lung cancer with mutated EGFR. N Engl J Med (2010) 362(25):2380–8. 10.1056/NEJMoa0909530 20573926

[5] Shi YK Wang L Han BH Li W Yu P Liu YP First-line icotinib versus cisplatin/pemetrexed plus pemetrexed maintenance therapy for patients with advanced EGFR mutation-positive lung adenocarcinoma (CONVINCE): a phase 3, open-label, randomized study. Ann Oncol (2017) 28(10):2443–50. 10.1093/annonc/mdx359 28945850

[6] Sequist LV Yang JC Yamamoto N O’Byrne K Hirsh V Mok T Phase III study of afatinib or cisplatin plus pemetrexed in patients with metastatic lung adenocarcinoma with EGFR mutations. J Clin Oncol (2013) 31(27):3327–34. 10.1200/JCO.2012.44.2806 23816960

[7] Wu YL Cheng Y Zhou X Lee KH Nakagawa K Niho S Dacomitinib versus gefitinib as first-line treatment for patients with EGFR-mutation-positive non-small-cell lung cancer (ARCHER 1050): a randomised, open-label, phase 3 trial. Lancet Oncol (2017) 18(11):1454–66. 10.1016/S1470-2045(17)30608-3 28958502

[8] Mok TS Wu YL Ahn MJ Garassino MC Kim HR Ramalingam SS Osimertinib or Platinum-Pemetrexed in EGFR T790M-Positive Lung Cancer. N Engl J Med (2017) 376(7):629–40. 10.1056/NEJMoa1612674 PMC6762027 27959700

[9] Rotow J Bivona TG Understanding and targeting resistance mechanisms in NSCLC. Nat Rev Cancer (2017) 17(11):637–58. 10.1038/nrc.2017.84 29068003

[10] Sorber L Zwaenepoel K Deschoolmeester V Van Schil PE Van Meerbeeck J Lardon F Circulating cell-free nucleic acids and platelets as a liquid biopsy in the provision of personalized therapy for lung cancer patients. Lung Cancer (2017) 107:100–7. 10.1016/j.lungcan.2016.04.026 27180141

[11] Gristina V Malapelle U Galvano A Pisapia P Pepe F Rolfo C The significance of epidermal growth factor receptor uncommon mutations in non-small cell lung cancer: A systematic review and critical appraisal. Cancer Treat Rev (2020) 85:101994. 10.1016/j.ctrv.2020.101994 32113081

[12] Qin J Wang J Lin X Wang J Xiong Z Wang R Erlotinib Resistance with EGFR L858R/Y891D Double Mutation in a Patient with Non-Small Cell Lung Cancer. J Thorac Oncol (2019) 14(4):e65–e8. 10.1016/j.jtho.2018.12.031 30922578

[13] Riley DS Barber MS Kienle GS Aronson JK von Schoen-Angerer T Tugwell P CARE guidelines for case reports: explanation and elaboration document. J Clin Epidemiol (2017) 89:218–35. 10.1016/j.jclinepi.2017.04.026 28529185

[14] Wang Z Yang JJ Huang J Ye JY Zhang XC Tu HY Lung Adenocarcinoma Harboring EGFR T790M and In Trans C797S Responds to Combination Therapy of First- and Third-Generation EGFR TKIs and Shifts Allelic Configuration at Resistance. J Thorac Oncol (2017) 12(11):1723–7. 10.1016/j.jtho.2017.06.017 28662863

[15] Westover D Zugazagoitia J Cho BC Lovly CM Paz-Ares L Mechanisms of acquired resistance to first- and second-generation EGFR tyrosine kinase inhibitors. Ann Oncol (2018) 29(suppl_1):i10–i9. 10.1093/annonc/mdx703 PMC6454547 29462254

[16] Kis O Kaedbey R Chow S Danesh A Dowar M Li T Circulating tumour DNA sequence analysis as an alternative to multiple myeloma bone marrow aspirates. Nat Commun (2017) 8:15086. 10.1038/ncomms15086 28492226 PMC5437268

[17] Hata AN Niederst MJ Archibald HL Gomez-Caraballo M Siddiqui FM Mulvey HE Tumor cells can follow distinct evolutionary paths to become resistant to epidermal growth factor receptor inhibition. Nat Med (2016) 22(3):262–9. 10.1038/nm.4040 PMC4900892 26828195

[18] Masood A Kancha RK Subramanian J Epidermal growth factor receptor (EGFR) tyrosine kinase inhibitors in non-small cell lung cancer harboring uncommon EGFR mutations: Focus on afatinib. Semin Oncol (2019) 46(3):271–83. 10.1053/j.seminoncol.2019.08.004 31558282

